# Analyzing animal behavior via classifying each video frame using convolutional neural networks

**DOI:** 10.1038/srep14351

**Published:** 2015-09-23

**Authors:** Ulrich Stern, Ruo He, Chung-Hui Yang

**Affiliations:** 1 Independent researcher, Durham, NC 27705; 2 Dept. of Neurobiology, Duke University, Durham, NC 27710.

## Abstract

High-throughput analysis of animal behavior requires software to analyze videos. Such software analyzes each frame individually, detecting animals’ body parts. But the image analysis rarely attempts to recognize “behavioral states”—e.g., actions or facial expressions—directly from the image instead of using the detected body parts. Here, we show that convolutional neural networks (CNNs)—a machine learning approach that recently became the leading technique for object recognition, human pose estimation, and human action recognition—were able to recognize directly from images whether *Drosophila* were “on” (standing or walking) or “off” (not in physical contact with) egg-laying substrates for each frame of our videos. We used multiple nets and image transformations to optimize accuracy for our classification task, achieving a surprisingly low error rate of just 0.072%. Classifying one of our 8 h videos took less than 3 h using a fast GPU. The approach enabled uncovering a novel egg-laying-induced behavior modification in *Drosophila*. Furthermore, it should be readily applicable to other behavior analysis tasks.

Understanding the neural mechanisms that control animal behavior often requires recording animals during behavioral tasks and then analyzing the videos. Human analysis of the videos is both highly labor-intensive and possibly error-prone, making automation very desirable and often critical to achieve acceptable throughput. To address this problem, multiple software systems for analyzing animal behavior have been developed, either for a particular model organism such as *Drosophila*^1^,^2^,^3^,^4^,^5^ or mice^6^,^7^, or less commonly, for multiple species^8^,^9^,^10^. For a comprehensive and up-to-date review, see Anderson and Perona^11^. Such software systems first—during the tracking phase—analyze each frame of the video individually, detecting the main parts of the bodies and possibly the appendages, and then use the detected parts as basis for further behavior analysis. But the systems rarely attempt to recognize “behavioral states”—e.g., actions or facial expressions—directly from images (“holistically”); instead, the systems recognize behavioral states from the explicitly detected parts.

Convolutional neural networks (CNNs)^12^, a machine learning approach, are a promising technique for recognizing behavioral states directly from images. CNNs are, by a large margin, the most successful technique in object recognition; they won the ImageNet Large Scale Visual Recognition Challenge 2012 (ILSVRC2012)^13^,^14^—where the task was to recognize 1000 different object classes including, e.g., mushroom, leopard, and motor scooter—by a decisive margin^15^, and recent progress has gotten them to close-to-human-level performance on ImageNet^14^. CNNs also recently became the leading technique for both 2-dimensional and 3-dimensional human pose estimation, where the goal is to estimate the positions of several “key” joints such as shoulders, elbows, and wrists^16^,^17^,^18^,^19^,^20^. While CNNs could hence likely improve body part detection in traditional tracking systems, our goal was to recognize behavioral states directly from images. Lastly and most relevant, CNNs recently became the leading technique for human action recognition on the PASCAL VOC2012 action classification challenge^21^, where the task is to recognize ten actions such as jumping, walking, and reading^22^,^23^. For action recognition it is often critical to recognize differences in pose and in the relationship between the actor and the environment (context); in contrast, in object recognition it is important to learn that different poses and contexts do not change the class of the object. Despite CNNs’ potential, however, to our knowledge the latest techniques have not been applied to behavior analysis in neuroscience.

Here, we describe how we applied CNNs to recognizing whether *Drosophila* were “on” egg-laying substrates (standing or walking) or “off” (not in physical contact with the substrate) for each frame of our videos. We achieved classification error rates on this 2-class problem of just 0.072% (i.e., 99.93% correct classification). The low error rate was surprising to us given, e.g., that the best reported mean average precision on the 10-class VOC2012 action classification challenge is 70.5%^22^; our average precision is 99.99906%. Classifying one of our 8h  videos, which has 216,000 frames and 2 flies per frame, typically took less than 2.5 h using an NVIDIA GTX TITAN GPU. We shed light on the techniques we used, ranging from generating labeled (“ground truth”) images for training, the architecture of the nets, training them, measuring their performance, and applying them to video analysis. Moreover, applying CNNs to our videos uncovered a novel egg-laying-induced behavior modification in *Drosophila* females that was difficult to ascertain with a conventional tracking approach. None of our techniques is specific to *Drosophila* egg-laying, and the same approach should be readily applicable to other species and behavior analysis tasks. Our data and code, which uses and extends Krizhevsky’s cuda-convnet code that won ILSVRC2012 (http://code.google.com/p/cuda-convnet/), is available on Google Code as project yanglab-convnet (http://code.google.com/p/yanglab-convnet/).

## Results

**Figure 1:**
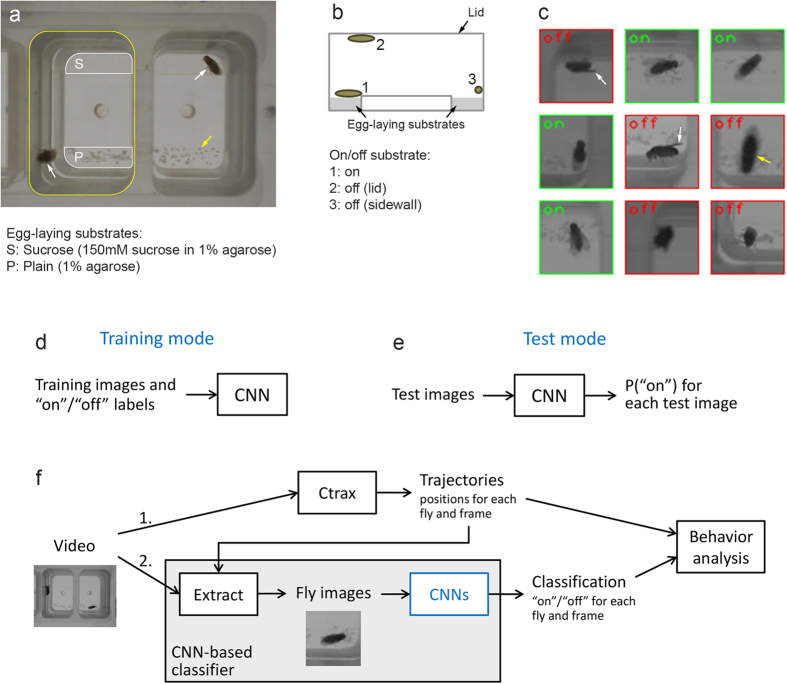
The problem addressed with neural networks and bird’s-eye view of the methodology. (**a**) Sample frame from one of our videos, showing two chambers with one fly in each chamber (white arrows). For the left chamber, the top edge of the chamber sidewall is outlined in yellow, and the two egg-laying substrates at the bottom of the chamber are outlined in white. The yellow arrow points to one of the many eggs laid on the plain substrates. (**b**) Schematic of the cross section of one chamber. We record through the lid with a camera above the chamber. In all three positions (1, 2, 3) shown, the fly would appear *over* the egg-laying substrate in a video, but it is *on* it only in position 1. (**c**) Sample fly images where the flies appears *over* the egg-laying substrate, with green and red labels indicating whether they are actually “on” or “off” the substrate. Flies on the sidewall often show a characteristic wing shape (white arrows). Flies on the lid are closer to the camera and appear larger and out of focus (yellow arrow). The fly images here have lower resolution than (**a**) since we reduced resolution for tracking and classification. (**d**) CNN in training mode. See text for details. (**e**) CNN in test mode. For each image the CNN is presented with, it calculates P(“on”), the probability the image is “on” substrate. We considered the net to classify an image as “on” if and only if P(“on”) ≥0.5. (**f**) Overview of our video analysis, which employs both positional tracking by Ctrax and classification by a CNN-based classifier. The classifier uses position information in two ways: first, to extract fly images from the full frame and, second (not shown), it needs to classify only if the fly is *over* the substrate (the fly is guaranteed to be “off” substrate otherwise). In our videos, the flies were over the substrate in typically about half of the frames.

We use *Drosophila* egg-laying site selection as model system to study the behavioral and circuit mechanisms that underlie simple decision-making processes, taking advantage of the system’s robustness and genetic tractability^24^,^25^,^26^,^27^,^28^,^29^,^30^. We previously discovered that *Drosophila* females prefer to lay their eggs on a sucrose-free (plain) substrate over a sucrose-containing one in two-choice chambers we designed (Fig. 1a)^28^,^30^. To study the decision process in more depth, we wanted to examine how females explore the two substrates before they execute each egg-laying decision. Conventional positional tracking can determine the (x, y)-position of the female at a given moment and hence whether the female is “over” one or the other substrate. But knowing whether the female is “over” the substrate does not allow us to distinguish whether it is truly in physical contact with the substrate (“on substrate”) or standing on sidewall or lid instead (“off substrate”) (Fig. 1b,c), a critical parameter for assessing females’ substrate exploration pattern.

We initially tried to automate the “on”/“off” substrate classification by using information that Ctrax^2^, the tracking software we used, provides in addition to the (x, y)-position of the fly. Ctrax fits an ellipse around the fly, and we used the lengths of the two axes of this ellipse to classify whether flies “over” the substrate were “on” or “off” (details not shown). But this approach did not perform well. First, due to (somewhat) differing brightness levels and focal planes in our videos, the classifier thresholds needed to be tuned for each video separately to achieve reasonable performance (about 3–5% error rate) on that video, hence requiring that humans generate labeled images to tune the classifier for each new video. Second, certain mutant flies (*TH* > *dTrpA1*) that we wanted to investigate spread their wings much more frequently than wild type, causing poor ellipse fits, which, in turn, not even allowed reasonable performance.

Since CNNs had the potential to work much better on our videos and learn to handle their variance, we tried them and got to about 1% error rate relatively quickly. We hence decided to switch to trying to optimize them instead of trying to improve our initial classifier. We were initially hoping to optimize the CNNs’ performance until it is comparable to the error rate of about 0.2% exhibited by the three *Drosophila* researchers in our lab who generated labeled images (see discussion why the “true” human error rate is lower than this 0.2%), but got to as little as 0.072% using relatively straightforward techniques.

Before we describe in detail how we used CNNs to automate the “on”/“off” substrate classification, we give a bird’s-eye view of CNNs. A CNN can operate in two different modes: training and test. In training mode, the CNN is presented (repeatedly) with a set of training images along with the correct class (label)—“on” or “off” in our case—for each image (Fig. 1d) and uses this information to learn to classify. In test mode, learning is turned off and the CNN’s performance is evaluated on a set of test images (Fig. 1e).

An overview of where we used CNNs in our approach to analyzing fly videos is shown in Fig. 1f. Note that the behavior analysis relies both on positional information from Ctrax and on classification information from our CNN-based classifier. The positional information is also used to extract (crop) the small “fly images” (with the fly in the center) (Fig. 1c) from the full frame (Fig. 1a).

### CNN architecture

**Figure 2:**
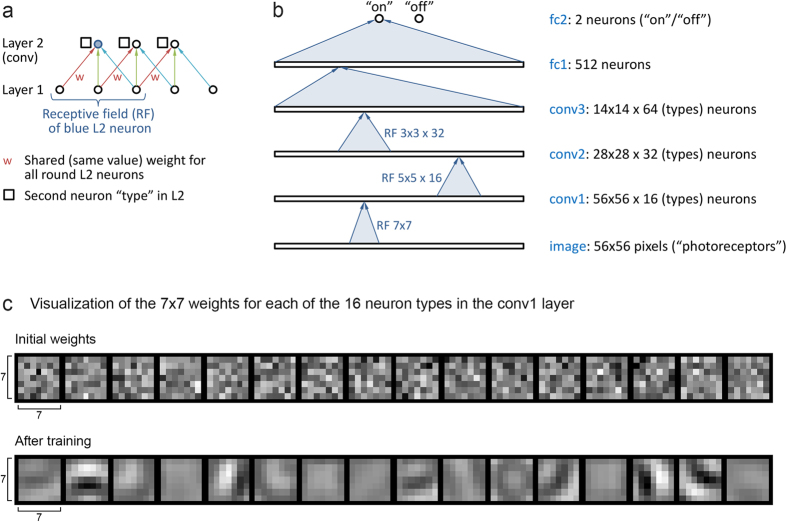
Architecture of the net we used. (**a**) Sample two-layer neural net where layer 2 is convolutional, showing the key ideas of convolution. First, the receptive field of each layer 2 neuron (or unit) is limited to a local subset of layer 1 neurons. Second, there are multiple types of layer 2 neurons (here round and square; the connections for square are not shown). Third, all neurons of the same type in layer 2 share weights; e.g., all red connections have the same weight value, and during learning, the weights of the red connections are adjusted identically. (**b**) Schematic of the architecture we used, with three convolutional and two fully-connected (fc) layers. Like in the architecture that won ILSVRC2012, each convolutional layer was followed by a max-pooling layer (not shown) with overlapping pooling, and we used rectified linear units^15^,^42^. For full details of the architecture, see the layer definition file in project yanglab-convnet on Google code. (**c**) Visualization of the weights learned by one of our CNNs for the conv1 layer. See text for details.

The key feature of the architecture of CNNs is convolution (Fig. 2a). A convolutional layer in a CNN is defined by two properties. First, each of its neurons has a limited (“local”) receptive field. Second, the (incoming) weights are shared (have the same values) among its neurons. In addition, a convolutional layer typically uses multiple neuron types, with each type having separate weights from the other types. For an example, consider the conv1 layer of the actual CNN architecture we used (Fig. 2b). The conv1 layer has 16 neurons (types) “above” each of the 56 × 56 pixels of the image. Each neuron has a receptive field of 7 × 7 pixels. The 7 × 7 weights for a particular type are shared among all 56 × 56 neurons of that type and can hence be represented by a single 7 × 7 matrix. Since there are 16 types in the conv1 layer, all the weights for the conv1 layer can be represented by 16 7 × 7 matrices (Fig. 2c).

We arrived at the particular CNN architecture—i.e., number of layers, number of types and size of receptive fields for each layer, etc.—we chose (Fig. 2b) after some initial experiments (not shown) by choosing a middle ground between one of the sample architectures Krizhevsky included in cuda-convnet and the architecture he used to win ILSVRC2012^15^. Generally, modifying the architecture can be used to optimize CNN performance; we did not experiment with the architecture further since we achieved a very low error rate with this architecture.

### Generating labeled images for training and test

**Figure 3:**
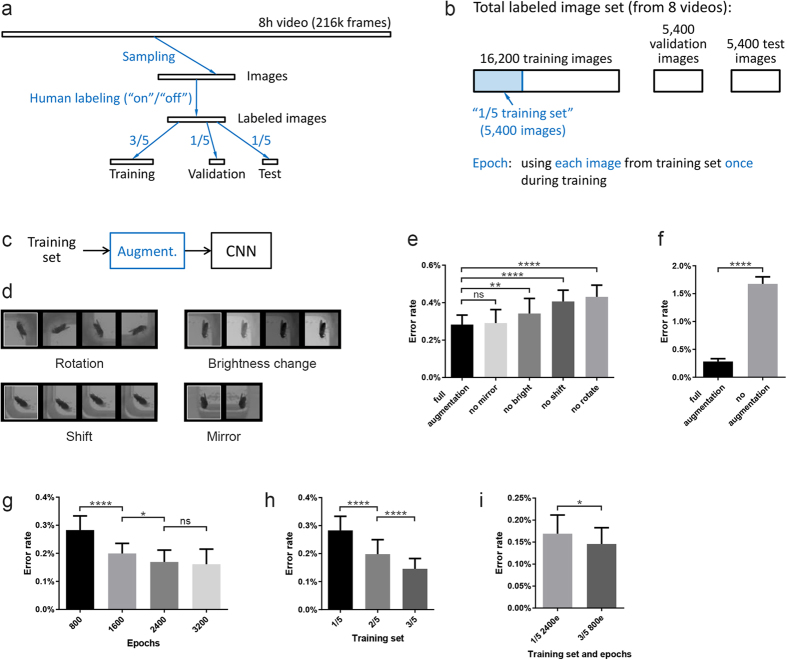
How we trained the nets. (**a–b**) Overview of how we created “on”/“off” labeled fly images for training and testing the CNNs. See text for details. (**c**) Overview of how we augmented the data. See text for details. (**d**) Sample images for the four image transformations we used to augment the data. (**e**) Data augmentation reduces the error rate. The control has all four image transformations enabled during training (“full augmentation”), the other bars show cases with only three of the four transformations enabled (i.e., one transformation disabled). All nets were trained for 800 epochs on the 1/5 training set. n = 30 nets per bar, bars show mean with SD, also for the following panels. One-way ANOVA followed by Dunnett’s test, p < 0.0001. (**f**) Full augmentation vs. no augmentation. All nets were trained on the 1/5 training set. For full augmentation, 800 epochs were used. For no augmentation, 400 epochs were used since the error rate was less for 400 epochs (mean 1.677%) than for 800 epochs (mean 1.740%), which is due to earlier overfitting (see text for Fig. 3g) for no augmentation. Welch’s t-test, p < 0.0001, two-tailed. (**g**) Additional training, up to a point, reduces the error rate. See text for why this is generally the case. All nets were trained on the 1/5 training set with full augmentation. One-way ANOVA followed by Šídák’s test, p < 0.0001. (**h**) Increasing the size of the training set reduces the error rate when the number of epochs is constant. The numbers of images in the 1/5, 2/5, and 3/5 training sets are 5,400, 2*5,400, and 3*5,400, respectively. All nets were trained for 800 epochs with full augmentation. One-way ANOVA followed by Šídák’s test, p < 0.0001. (**i**) The 3/5 training set reduced the error rate compared to the 1/5 training set when the total number of images seen by the CNN during training was constant. “1/5 2400e” denotes training on the 1/5 training set for 2400 epochs, etc. All nets were trained with full augmentation. Welch’s t-test, p = 0.026, two-tailed.

For training and testing the CNNs, we created a large number of fly images that were “labeled” with the correct class (“on”/“off”) by humans (Fig. 3a, Supplementary Fig. S1a). The instructions to the humans were to label “on”/“off” as well as possible and tag the image in case they were not sure about the label. To minimize labeling errors, we labeled each image independently by three humans and used only images for which all three humans agreed and none of them had tagged the image. We generated a total of 27,000 labeled images this way from eight videos, and we partitioned the images into three sets: training, validation, and test (Fig. 3b). We used the validation set to measure the error rate of the CNNs while we optimized their performance (“tuned hyperparameters”), while we used the test set only once we settled on a “best net”. The total time required for labeling was only about 10 hours per human. We chose half of the videos for labeling among videos of the mutants that spread their wings much more frequently. We also noticed that randomly sampling video frames for labeling can lead to highly correlated fly images if flies rest for longer periods (Supplementary Fig. S1b), which we addressed by modifying the sampling process to resample until a sample is found for which its normalized cross-correlation^31^ with each of the 6 already chosen samples “surrounding” it (in frame order) is smaller than 0.95.

### Training and techniques to improve performance

During training, the CNNs were presented a set of labeled training images multiple times, and backpropagation^32^ with stochastic gradient descent was used to adjust the weights to enable the nets to learn “on” vs. “off” substrate. Using each image from the training set once during training is called an epoch. Using the GTX TITAN GPU, training one CNN on our “1/5 training set” with 5,400 images (Fig. 3b) for 800 epochs (i.e., using the same 5,400 images 800 times) took about 17.5 minutes. We typically trained 30 different CNNs to be able to calculate statistics of their performance. (The nets differed since we used randomized initial weights and data augmentation, discussed next.) See Methods section for more details on the training.

A well-known technique to increase the amount of training data—called data augmentation—is to transform the data without changing the class it falls into (Fig. 3c,d). E.g., slightly shifting the fly image does not change whether the fly image is “on” or “off” substrate. While this is “obvious” to humans, it is something the CNN “needs” to learn. Each time a fly image was selected for training from the training set, it was first randomly transformed using all four transformations (Fig. 3d and Methods section), so many different versions of each training set image would be used when training over, e.g., 800 epochs. Note that this “dynamic transformation” approach effectively increased the size of the training set without the need to store a larger, augmented training set. As expected, data augmentation strongly improved performance (Fig. 3e,f).

The time required to train a CNN for a certain number of epochs (training time) essentially stayed the same with data augmentation. The training time is proportional to the total number of images seen by the CNN during training, which equals the size of the training set multiplied by the number of epochs, and the image transformations for data augmentation increased the time required per image seen only slightly.

We also experimented with the number of epochs used for training and the size of the training set (Fig. 3g–i). Typically, when increasing the number of epochs (and using a training set that is different from the validation set on which the error rate is calculated), the error rate initially drops, reaches its minimum, and then starts to increase (not shown). The increase is due to overfitting^33^, where the net picks up details on the training set that are not relevant for the task. For the 1/5 training set, we stopped increasing the number of epochs at 3200 epochs, where additional training seemed to no longer improve performance and the total training time for 30 nets reached 33.8 hours (Fig. 3g). Increasing the size of the training set from 1/5 to 3/5 of the total labeled images while training for 800 epochs improved performance (Fig. 3h), but this was at least partly due to the fact that for a constant number of epochs, a larger training set results in the CNN’s seeing more images during training. When we increased the number of epochs for the 1/5 training set to 2400 to match the total number of images seen during training on the 3/5 training set for 800 epochs, the latter showed only a small improvement in performance over the former (Fig. 3i). But we expect increasing the number of epochs for the 3/5 training set from the 800 used here would have allowed us to reduce the error rate similar to what we observed for the 1/5 training set (Fig. 3g). We did not pursue this since the total training time for 30 nets on the 3/5 training set for 800 epochs was 25.7 hours and the techniques discussed next strongly lowered error rates to levels good enough for our application.

### Techniques to improve performance used during testing

**Figure 4:**
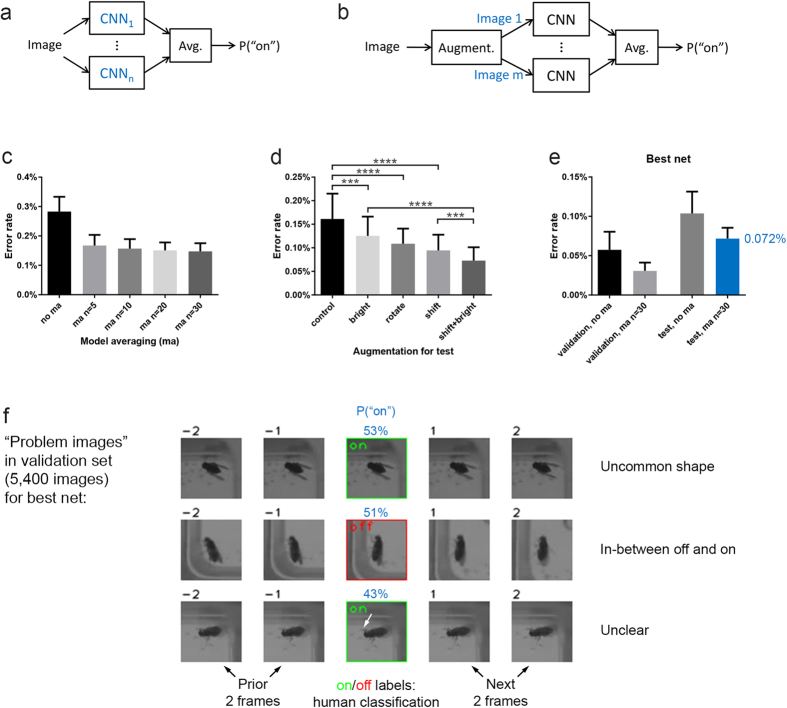
Applying the nets to classifying single images. (**a**) Overview of model averaging using n models (CNNs). See text for details. (**b**) Overview of augmentation for test using m images. See text for details. (**c**) Model averaging (ma) reduces the error rate. “ma n = 5” denotes model averaging using 5 models, etc. Each bar is based on the same 30 nets, and the bootstrap^43^ is used to estimate the mean and variance of model averaging by repeatedly (500 times) sampling with replacement the n nets used for model averaging from the 30 nets. All nets were trained for 800 epochs on the 1/5 training set with full augmentation. Bars show mean with SD, also for the following panels. No statistical tests were run since the bootstrap gives only *estimates* of the error rate distributions. (**d**) Augmentation for test reduces the error rate. All nets were trained for 3200 epochs on the 1/5 training set with full augmentation. Same n = 30 nets for the five bars, repeated measures ANOVA with Geisser-Greenhouse correction followed by Šídák’s test, p < 0.0001. (**e**) Validation and test error rates for our “best nets”, both without and with model averaging. The best nets were trained using full augmentation on the 3/5 training set for 800 epochs and used shift and brightness change augmentation during testing. Same 30 nets for all four bars, model averaging estimated using the bootstrap. (**f**) Validation set images that were difficult for the best nets. Model averaging of 20 nets and 500 bootstrap repeats were used to determine difficult images. The images are shown with the 2 prior and 2 next frames in the video, which can help humans to assess the cases. See text for a discussion of the first two cases. In the last case, it is unclear why the nets tended to make a mistake. It is possible the darker area close to the head of the fly (white arrow) was mistaken for the characteristic “sidewall wing” shape, an error humans would clearly not make.

We employed two techniques during testing that strongly improved performance. First, since we typically trained 30 different nets, we could present the image to multiple of the nets and average their “on” probabilities, a simple form of model averaging (Fig. 4a). We typically got the best performance presenting the images to all 30 nets (Fig. 4c). But using just 5 nets was typically close in error rate to 30 nets, while requiring 6-fold less processing.

The second technique that strongly improved performance was to use data augmentation (Fig. 3d) also during testing, by presenting multiple transformed versions of the image to the *same net* and then averaging the “on” probabilities (Fig. 4b). Unlike when we used data augmentation for training, we did not randomize the transformations. For example, for brightness change, we presented the net with 4 versions—original, brighter, darker, and increased contrast (Fig. 3d). See Methods section for more details. Combining shift and brightness change strongly reduced the error rate for our task (Fig. 4d).

### Our best nets

We then tried to create a well-performing net using what we learned during our experiments to optimize training and testing. The resulting “best nets” were trained using full augmentation (i.e., all four image transformations enabled) on the 3/5 training set for 800 epochs and used shift and brightness change augmentation during testing. With model averaging of 30 models, the error rate was just 0.031% on the validation set (1.66 errors on 5,400 images) and 0.072% on the test set (Fig. 4e).

We examined the three validation set images that were difficult for the best nets (Fig. 4f). Two of the cases appear to be easy to explain — one had the fly in uncommon shape that was likely rare during training and one was in-between “off” and “on” and should likely have been tagged by humans as “not sure”. We also examined the eight test set images that were difficult for the best nets (not shown) and noticed several “sidewall” cases like the one shown in Supplementary Figure S2b (see next subsection). “Sidewall” cases can explain the majority of the difference between validation and test error rates.

### Applying nets to videos

**Figure 5:**
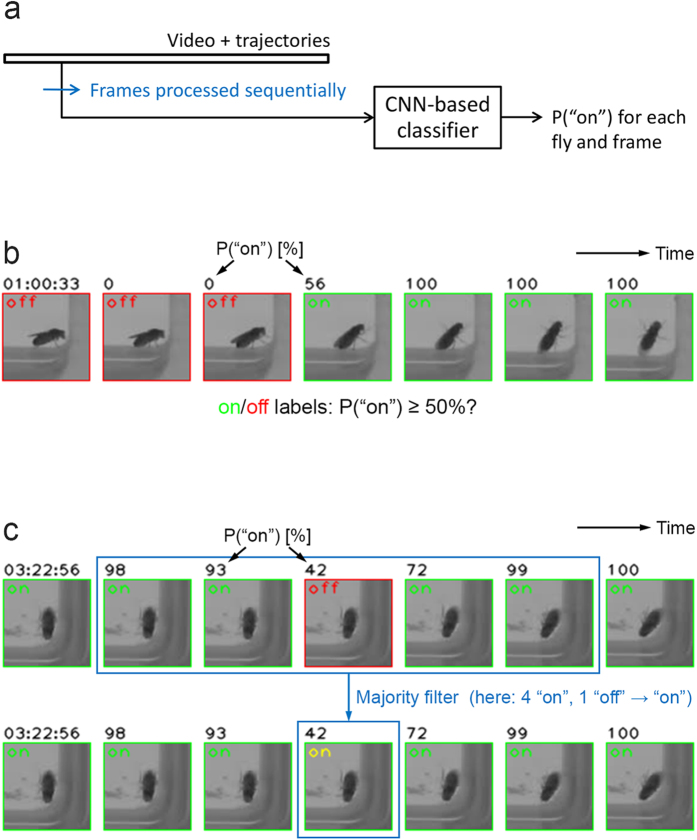
Applying the nets to videos. (**a**) Overview of how we applied the nets to videos. See text for details. (**b**) Fly images sequence from consecutive frames classified by the nets. The fly walks from the sidewall onto the substrate in this case. Unlike earlier in the paper, the labels no longer represent human classification but now represent the nets’ classification. (**c**) Fly image sequence where the majority filter fixed a mistake of the nets. The same sequence is shown before (top) and after (bottom) the fix, with the new (correct) label in yellow. See text for details of the majority filter.

To apply the nets to videos, we sequentially processed each frame (Fig. 5a,b). We used both model averaging and augmentation for “test” to improve performance. Combining both techniques resulted in many “on” probability calculations for each fly image. E.g., we used 30 nets and presented each with 20 versions (5 shifts × 4 brightness levels) of each image, resulting in 600 “on” probability calculations for each image. Nonetheless, we could process about 27 images per second using a GTX TITAN GPU, allowing our 8 h videos to be classified in typically less than 2.5 h each. If we had been willing to accept higher error rates, we could have processed our videos up to 600 times faster.

Examining the frames before and after a frame to be classified is often helpful for humans. We implemented this idea in a simple majority filter that replaces the “on”/“off” value of each frame with the majority “on”/“off” vote of the 5 consecutive frames with the current frame in the center (Fig. 5c). Note that the majority filter correctly preserves “on”/“off” transitions like the one in Fig. 5b. But the filter can make mistakes, e.g., for visits to the substrate for just two frames (Supplementary Fig. S2a), causing such visits to be missed in an analysis based on the output of the filter. Two-frame visits lasted for only about 0.3 s in our case (our frame rate was 7.5 fps) and for our analysis, missing them seemed acceptable.

We manually checked the performance of the nets in the times before egg-laying events for several videos. In the process, we noticed some mistakes, which were almost all false negatives (Supplementary Fig. S2b). But the mistakes seemed rare enough that further improvements in classification performance seemed not required for our behavior analysis.

### *Drosophila* females show egg-laying-induced increased sucrose attraction

**Figure 6:**
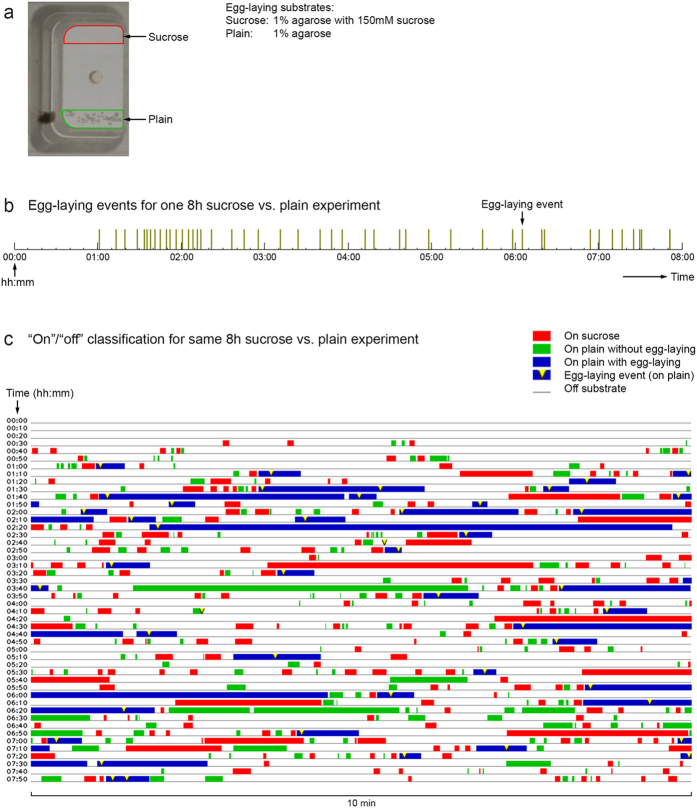
“On”/“off” classification for one 8 h sucrose vs. plain experiment. **(a)** Chamber image with egg-laying substrates outlined. (**b**) Visualization of the egg-laying events for one 8 h sucrose vs. plain experiment. The fly laid 46 eggs, all on the plain substrate, during the 8 hours. (**c**) “On”/“off” classification for the same 8 h sucrose vs. plain experiment.

We next used our nets to investigate the substrate exploration pattern of females as they laid eggs in our *sucrose vs. plain* decision chambers over 8 hours (Fig. 6a). Each female typically deposited 50 or more eggs in an 8-hour recording session and laid one egg at a time (virtually all on the plain substrate) with no fixed interval between consecutive egg-laying events (Fig. 6b). Applying our nets to the videos provided us with complete descriptions of females’ substrate exploration patterns for many 8-hour sessions, which, in turn, allowed us to begin scrutinizing how they behave before executing each egg-laying decision (Fig. 6c).

**Figure 7:**
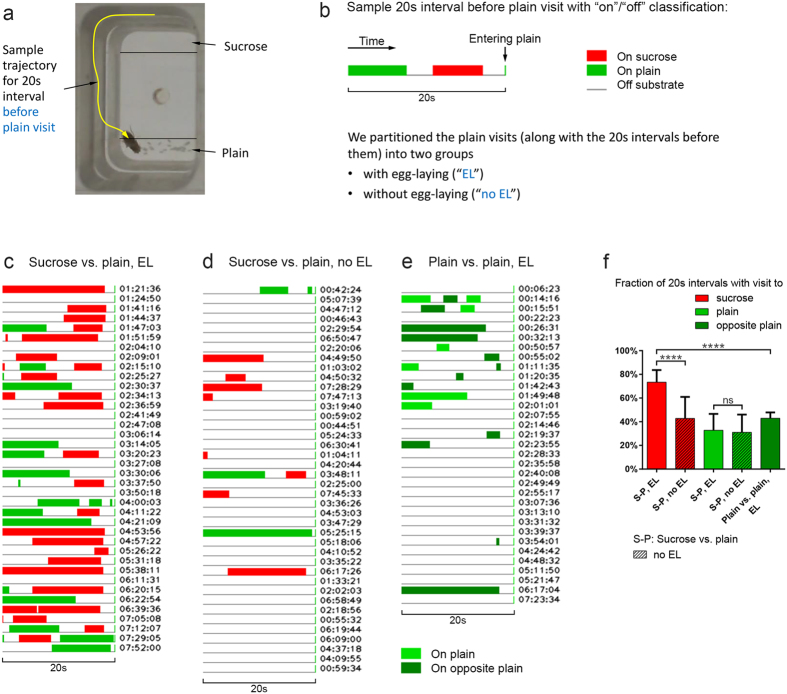
*Drosophila* females show increased sucrose contact prior to egg-laying. (**a**) Chamber image with sample trajectory for 20 s interval before visit to plain substrate. For this trajectory, the fly is “off” substrate for all frames, mostly on the sidewall. (**b**) Sample 20 s interval before plain visit with “on”/“off” classification. The green line at the end of the interval represents the first frame of the plain visit following the interval. During this plain visit, the female may or may not lay eggs. (**c–d**) 20 s intervals before plain visits *with* (**c**) and *without* (**d**) egg-laying for sucrose vs. plain assay. All intervals are from an 8 h video. The egg-laying times were manually annotated and are given next to the intervals in (**c**). The intervals for (**d**) were randomly chosen among the plain visits without egg-laying. (**e**) 20 s intervals before plain visits with egg-laying for *plain vs. plain* assay. All intervals are from an 8 h video and represent about half of the egg-laying events—those laid on one of the two plain sites. About an equal number of eggs was laid on the other plain (“opposite plain”) site. The egg-laying times were manually annotated and are given next to the intervals. (**f**) Fractions of 20 s intervals with visit to sucrose, plain, or opposite plain for the three cases from (**c–e**). The 20 s intervals for the four sucrose vs. plain (S-P) bars are from 10 flies, each recorded for 8 hours, yielding 540 intervals before plain visits with egg-laying and 400 randomly chosen intervals before plain visits without egg-laying (40 per fly). The 20 s intervals for the plain vs. plain bar are from 5 flies, each recorded for 8 hours, yielding 340 intervals before plain visits with egg-laying. Same n = 10 flies for first four bars, n = 5 flies for last bar, repeated measures ANOVA with Geisser-Greenhouse correction followed by Šídák’s test, p < 0.0001, and Welch’s t-test, p < 0.0001, two-tailed. Using the Bonferroni correction to adjust for the additional comparison (t-test) does not change significance.

Visual inspection of the full 8-hour substrate exploration suggests that females show more physical contact with the sucrose substrate prior to each egg-laying event (Fig. 6c), which, in turn, suggests that emergence of egg-laying need may temporarily increase females’ behavioral attraction towards sucrose. To test this idea formally, we determined females’ contact with the sucrose substrate in the 20 s intervals before they entered the plain substrate to deposit an egg and compared those with 20 s intervals before plain visits without egg-laying (Fig. 7a,b). We found that sucrose visits were more common during 20 s intervals before plain visits with egg-laying (Fig. 7c) than during 20 s intervals before plain visits without egg-laying (Fig. 7d,f). In contrast, the occurrence of plain visits during 20 s intervals before plain visits with and without egg-laying is comparable (Fig. 7f). Moreover, for sucrose visits for which the next substrate visit was to plain, the average time from sucrose exit to entering plain was significantly shorter for the “egg-laying group” (Supplementary Fig. S3), suggesting sucrose visits that directly precede an egg-laying event are different from sucrose visits that do not. Lastly, to rule out the possibility that the high occurrence of sucrose visits before plain visits with egg-laying can be explained by a tendency to visit the *opposite* substrate before egg-laying, we investigated how *Drosophila* behave in case both of the two substrates are plain (*plain vs. plain* assay, where females show no egg-laying preference between the two plain sides). For *plain vs. plain*, visits to the opposite substrate were relatively uncommon during the 20 s intervals before plain visits with egg-laying (Fig. 7e,f).

Taken together, our inspection and analysis of multiple 8-hour substrate exploration patterns showed that when a female is getting ready to deposit an egg on the plain substrate, it will often visit the sucrose substrate first. This result suggests that the emergence of egg-laying need can temporarily enhance *Drosophila* females’ attraction for sucrose and demonstrates for the first time that a behavioral state other than food deprivation can motivate *Drosophila* to seek contact with sucrose, perhaps to feed.

## Discussion

We described how we used CNNs to classify with very low error rate whether *Drosophila* are “on” or “off” substrate for each frame of our videos, allowing us to uncover an enhanced attraction for sucrose induced by egg-laying need. Using the processing power of a modern GPU helped significantly by speeding up both training the nets—and hence our experiments to minimize the nets’ error rate shown in Figs 3 and 4—and using the nets to process videos frame by frame. We expect our approach to be broadly applicable to other behavior analysis tasks for two reasons. First, the task performed by the CNNs and where they excel—deciding which one of several classes an image falls into—is a natural task in behavior analysis. Sometimes this may not be obvious at first; while we wanted a solution to “whether the flies are on or off the substrate in our videos”, we initially did not think of this as an image classification problem. Second, none of the techniques we used is specific to *Drosophila* or the behaviors we are interested in—an important advantage of a machine learning approach over domain-specific code.

We have so far not addressed the question of how our best nets’ 0.072% error rate on the “on”/“off” substrate task compares to human performance. Human errors on the task fall into two classes—“true errors” that humans would make even if they were very careful and had unlimited time for each image and errors that are due to “sloppiness”. We found that on this task—which is relatively simple for humans and where one needs a large number (thousands) of images to measure performance—the clear majority of the human errors is due to sloppiness and measuring the true human error rate is very difficult. We do hence not present such measurement. But it is worth to point out that our nets found several errors in the labeled images, even though for each such image three researchers with a high level of experience with *Drosophila* and our videos had agreed on the classification and were sure (i.e., had not tagged the image to indicate “not sure”) but were wrong if one were to inspect the images that precede and follow the image in question or the video (Supplementary Fig. S4). Other researchers have recently reported achieving human-level performance using neural nets for, e.g., face recognition^34^ and handwritten digit recognition^35^.

While the nets processed the frames one at a time, one interesting area for future work is better exploiting the information from *sequences* of frames. First, this can lead to better image classification. E.g., “before and after” frames were shown in multiple figures in this paper to help humans better understand what class an image falls into (e.g., Fig. 4f, Supplementary Fig. S4). (“Before and after” frames are also the basis of the simple majority filter we used.) Second and more importantly, many interesting behaviors require analyzing sequences of frames. One approach to address this is using the nets’ frame-by-frame classification as an input *feature* for a tool like JAABA^9^, which focusses on detecting behaviors from sequences of features of frames. With the continuing advances in easily available processing power and CNN technology (e.g.,^36^,^37^,^38^,^39^), however, using neural nets to classify not single images but sequences of images (parts of videos) is starting to be practical^40^,^41^.

Lastly, CNNs have improved our ability to study the behaviors of egg-laying females. CNNs enabled a complete and accurate description of females’ substrate exploration pattern, which allowed us to quickly discover that egg-laying demand can temporarily increase *Drosophila’*s attraction for sucrose. To our knowledge, this is the first demonstration that a physiological state other than food deprivation can enhance females’ behavioral attraction for sucrose, and we speculate that perhaps consuming sugar before an energetically demanding act confers some benefit to the egg-laying females. The clear behavioral readout will allow us to investigate the circuit mechanism by which egg-laying demand modifies the sensory processing of sucrose, an important taste stimulus in *Drosophila*. In addition, it is worth pointing out that there might be yet-to-be-discovered “rules” of substrate exploration that *Drosophila* females employ before they execute their egg-laying decisions, and that the availability of many hours worth of substrate exploration patterns may permit an unsupervised learning approach to uncover such rules.

## Methods

### Preparation of flies to be assayed

Flies (*w1118*) were raised on molasses food and maintained with temperature set at 25 °C and humidity set at 60%. To assay flies for their egg-laying decisions, 35–40 freshly eclosed virgins of the appropriate genotypes and about 25 males of mixed genotypes were collected into a single food vial that was supplied with active wet yeast paste. The flies were left in the vial for about 5 days until the food in the vial became very chewed up by the larvae. At this point, females were well fed but deprived of egg-laying because the food was too soft and wet for them to lay more eggs on. Thus, they were ready to lay eggs when placed into our behavior chambers.

### Behavior analysis

To record the females as they explored and laid eggs, we mounted 4 webcams (Microsoft LifeCam Cinema) on top of an apparatus containing multiple egg-laying chambers^30^. Females were prepared as described earlier but we recorded behaviors of egg-laying females for 8 hours only. We used several software packages: CamUniversal for video acquisition, Avidemux for video conversion, and Ctrax for positional tracking. Individual egg-laying events were determined manually from the videos and the output of a custom-written egg-laying detector. “On”/“off” substrate classification was performed using a modified version of Krizhevsky’s cuda-convnet code and custom-written Python code (see “Software we developed” below). Custom-written Python code was also used to analyze the flies’ behavior based on their egg-laying times and position and classification information for each frame.

### Labeled image data

See also “Generating labeled images for training and test” in Results section. To be able to use Krizhevsky’s cuda-convnet code, we divided the 21,600 labeled training and validation images into 36 batches of 600 images each, and used the same data layout. The batches contained 92 × 92-pixel grayscale images, which were reduced to 56 × 56 pixels (matching Fig. 2b) during data augmentation. Having “extra size” images in the batches simplified, e.g., the shift image transformation, which was implemented simply as a crop from the larger images. All but one of the eight videos we used as source for images contributed four batches; e.g., the first video contributed batches 1–4. One of the videos—the one we thought to have the highest variance in wing shapes (one of the videos with *TH *>* dTrpA1* flies)—contributed eight batches. Our validation set was batches 4, 8, …, and 36, while our 1/5 training set, e.g., was batches 1, 5, …, and 33. We generated the 5,400 image test set separately (we initially used our validation set for both validation and test) from the eight videos in a similar fashion, resulting in 9 test batches.

### CNN training protocol

Our training “protocol” was inspired by Krizhevsky’s Methodology page for cuda-convnet. When we wanted to train for 800 epochs total, we trained for 400 epochs using weight learning rate (epsW) 0.001, 200 epochs using 0.0001, and 200 epochs using 0.00001. We automated the protocol using custom code to train (typically) 30 nets without human interaction, which made optimizing the performance of our nets much less labor-intensive. (Note that often the learning rate is lowered manually when the validation error rate stops improving^15^,^38^, but such points were not easy to see with our very low error rates.) For full details of the learning parameters, see the layer parameter file in project yanglab-convnet on Google code.

### Data augmentation during training

During training, we dynamically and randomly augmented the training set using four image transformations (Fig. 3c,d). For rotation, we randomly selected among angles that are multiples of 90°. For brightness change, we first calculated the minimum and maximum brightness values over all pixels in a given image. We then randomly selected new minimum and maximum values with certain constraints such as that the difference of the values does not decrease (to not reduce information) and used the new values to normalize the image. For shift, we randomly shifted in x- and y-direction independently by up to 4 pixels, resulting in 81 possibilities.

### Data augmentation for test

During testing, we used image transformations in non-randomized fashion (Figs 3d and 4d). For brightness change, we presented the net with 4 versions of each image—original, brighter, darker, and increased contrast. For rotation, we used 4 versions—original, 90°, 180°, and 270°. For shift, we used 5 versions—original, (2, 2), (2, −2), (−2, 2), and (−2, −2), with (., .) denoting the shift for x- and y-axis in pixels. For shift + brightness change, we used 20 versions—all combinations of the 5 shift and 4 brightness change versions.

### Software we developed

Here we summarize the software we developed for this research and which we made available as project yanglab-convnet on Google code. Our software has two parts. First, we extended Krizhevsky’s cuda-convnet with two additional image transformations (rotation and brightness change) for data augmentation. Second, we wrote several Python “scripts” for various aspects of the research. onSubstrate.py was used to generate the labeled image data via human labeling. autoCc.py automates the training of multiple nets using cuda-convnet and implements model averaging for assessing multiple nets. classify.py was used to classify the fly images in each frame of a fly video. analyze.py was used to analyze the flies’ behavior and created, e.g., Figs 6c and 7c–e.

### Statistical methods

We used GraphPad Prism 6 to perform statistical tests and significance level α = 0.05. The statistical tests we performed when we optimized CNN performance (Figs 3e–i and 4d) helped us decide which techniques were beneficial in our particular application of CNNs for the particular CNN architecture we chose. The performance improvement a particular technique enables will vary depending on the application. Current papers on neural networks typically do not report whether tests were used when optimizing the nets’ performance, and when the number of nets trained is reported, it is typically smaller than our default of n = 30 nets per group^15^,^38^,^39^.

## Additional Information

**How to cite this article**: Stern, U. *et al.* Analyzing animal behavior via classifying each video frame using convolutional neural networks. *Sci. Rep.* **5**, 14351; doi: 10.1038/srep14351 (2015).

## Supplementary Material

Supplementary Information
