# Supplementary material for: Analyzing animal behavior via classifying each video frame using convolutional neural networks
Source: Sci Rep. 2015 Sep 23;5:14351. doi: 10.1038/srep14351 (PMC4585819; doi:10.1038/srep14351)
Supplement: Supplementary Information [file srep14351-s1.pdf]

Supplementary information

Analyzing animal behavior via classifying each video frame using convolutional neural networks

Ulrich Stern, Ruo He, and Chung-Hui Yang

Corresponding authors: ulrich.stern@gmail.com and yang@neuro.duke.edu

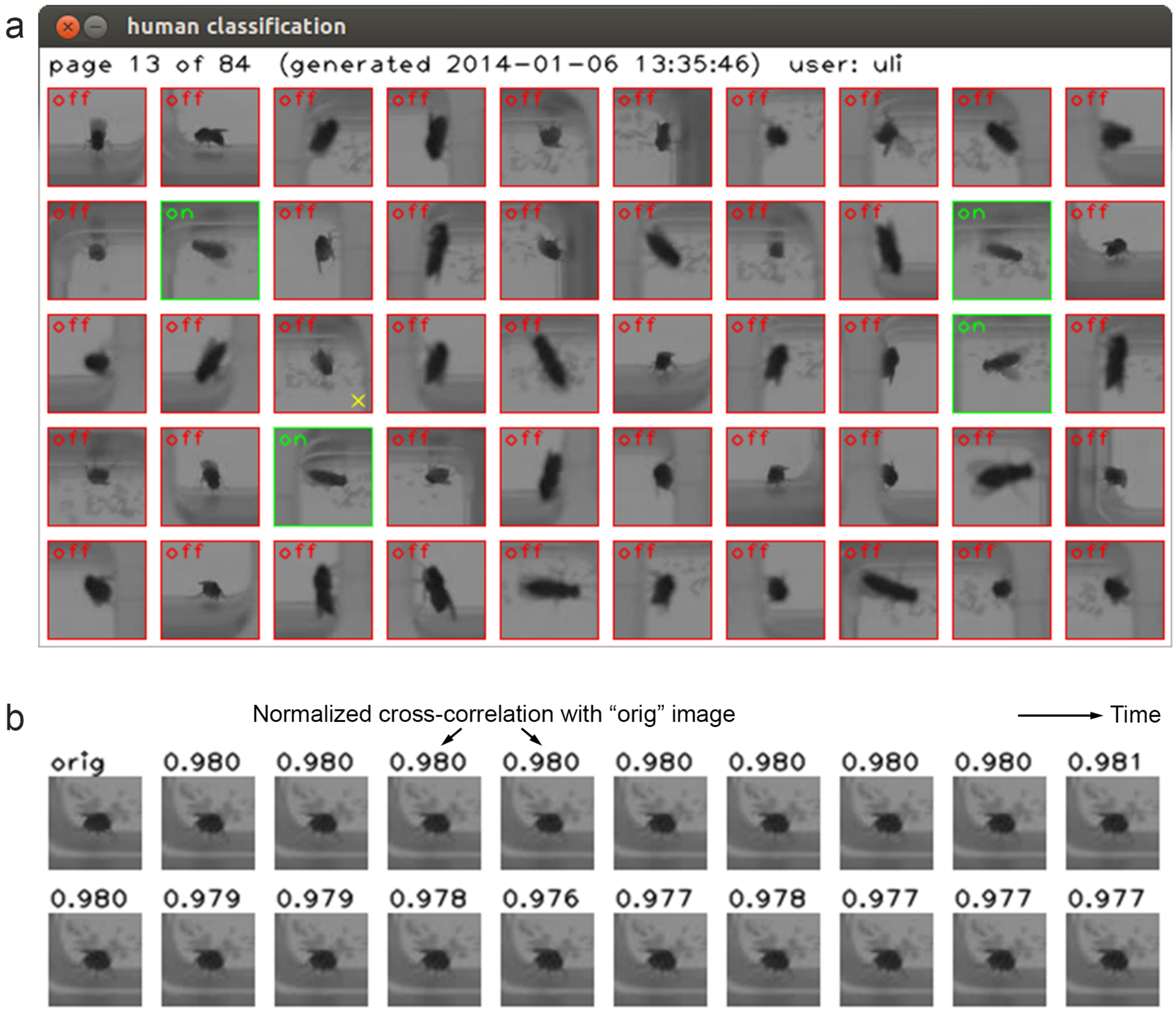

Supplementary Figure S1. Generating training and test data. (a) The tool we used for generating training and test data ("human labeling"). Each page had 50 fly images for labeling and typically took less than one minute to complete. The yellow "x" indicates that the image is "tagged," i.e., the human was not sure about the correct label. **(b)** Highly correlated fly images of a resting fly selected for labeling by randomly sampling video frames. The fly rested for about 24 minutes in this case. Only the first 20 images are shown; there were actually 65 of them.

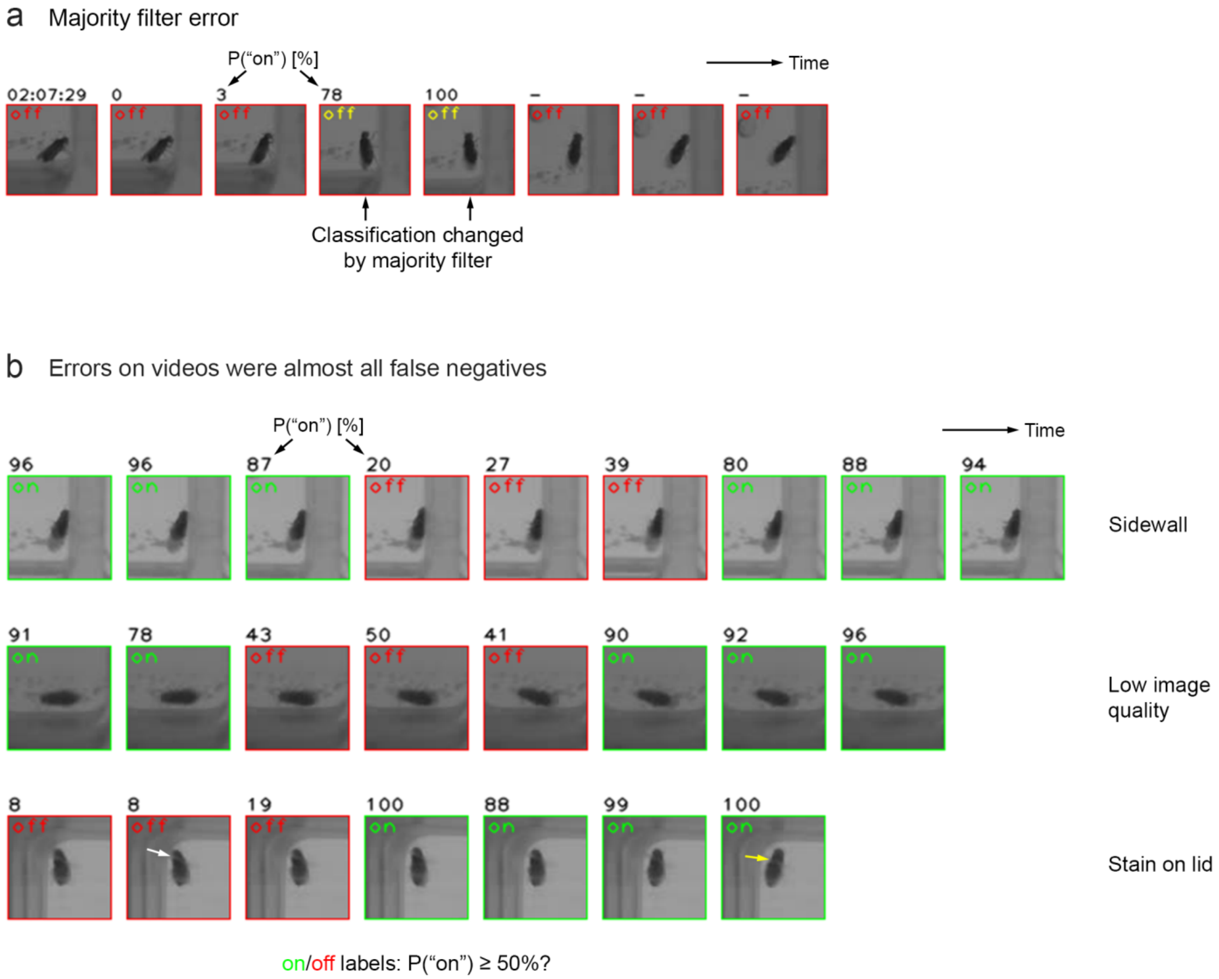

Supplementary Figure S2. Sample errors of the net on videos. (a) Fly image sequence where the majority filter introduced a mistake. The two yellow labels were changed to "off" by the majority filter. Note that the flies are "on" substrate in the two corresponding images, and that the nets had correctly classified the images giving "on" probabilities 78% and 100%, respectively. **(b)** Fly images sequences from consecutive frames for three sample errors. All "off" substrate classifications are incorrect and should have been "on." In the first case, the cause of the problem seems to be that the fly is partly hidden by the sidewall. Few such images made it into the training set since the sidewall problem occurs only for some videos and when it occurs, our rule to select only images for which three humans were certain in their classification tended to exclude them. Making "sidewall" images well represented in the training set would likely strongly reduce sidewall issues. For the last case, the cause of the problem seems a water stain on the lid of the chamber, which caused light gray lines over the fly (white arrow). The position of the lines on the fly body changes when the fly moves (yellow arrow). While the easy solution is to avoid stains on the lid, adding artificial stains during data augmentation or using a technique like dropout (35) on the input layer may make the nets less affected by stains.

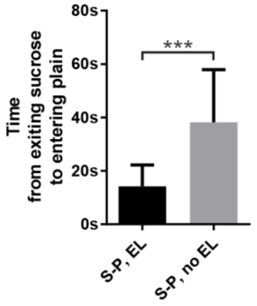

Supplementary Figure S3. Time from exiting sucrose to entering plain. Same flies and recordings as those used for sucrose vs. plain in **Figure 7f**. The bars are based on sucrose visits for which the next substrate visit was to plain, either with egg-laying on plain (424 sucrose visits) or without (391 randomly chosen sucrose visits, up to 40 per fly). Times were averaged for each fly. Same n = 10 flies for both bars, bars show mean with SD, ratio paired t-test, p = 0.0008, two-tailed.

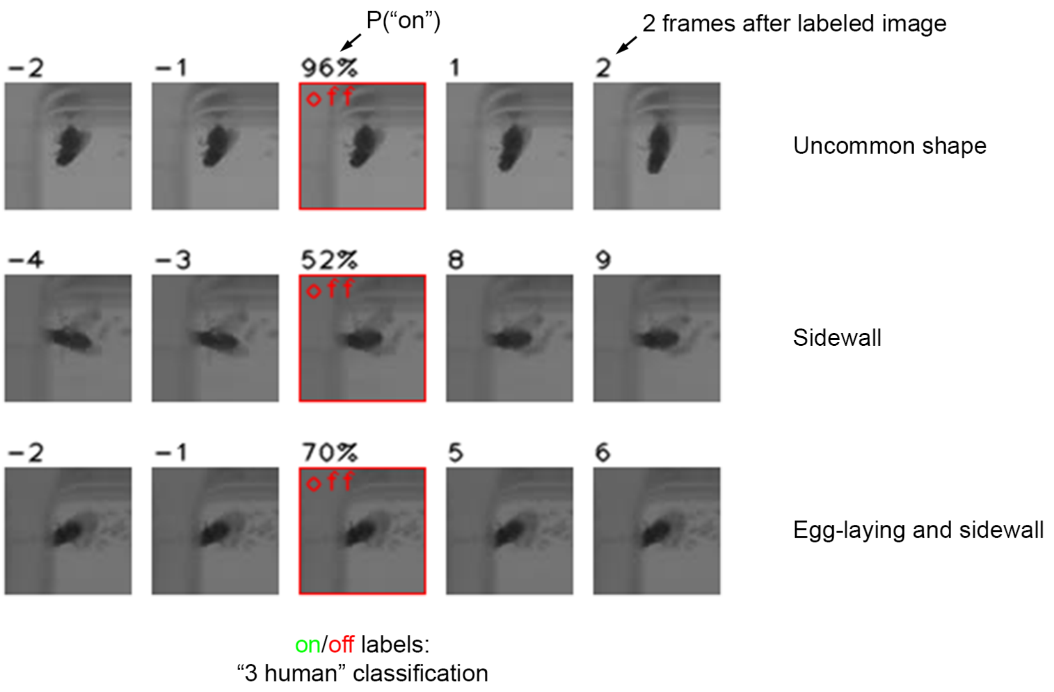

Supplementary Figure S4. Example human classification errors that the net caught. Three fly images where the "3 human" classification ("off") is incorrect but the nets were correct, including some "before and after" frames to make it easier to see the human errors. All three cases were also verified to be "on" substrate using the original videos that have higher resolution (not shown). Note that the types of problems humans had – uncommon shape and sidewall – were seen for the nets as well (**Fig. 4f**, **Supplementary Fig. S2b**).
